# An Auto-Regulating Type II Toxin-Antitoxin System Modulates Drug Resistance and Virulence in *Streptococcus suis*

**DOI:** 10.3389/fmicb.2021.671706

**Published:** 2021-08-12

**Authors:** Qibing Gu, Peijuan He, Dan Wang, Jiale Ma, Xiaojun Zhong, Yinchu Zhu, Yue Zhang, Qiankun Bai, Zihao Pan, Huochun Yao

**Affiliations:** ^1^College of Veterinary Medicine, Nanjing Agricultural University, Nanjing, China; ^2^Key Laboratory of Animal Bacteriology, Ministry of Agriculture, Nanjing, China; ^3^OIE Reference Laboratory for Swine Streptococcosis, Nanjing, China; ^4^College of Animal Science and Technology, College of Veterinary Medicine, Zhejiang A&F University, Hangzhou, China; ^5^Institute of Animal Husbandry and Veterinary Sciences, Zhejiang Academy of Agricultural Sciences, Hangzhou, China; ^6^College of Veterinary Medicine, Henan Agricultural University, Zhengzhou, China

**Keywords:** *Streptococcus suis*, toxin, antitoxin, drug resistance, virulence

## Abstract

Toxin-antitoxin (TA) systems are ubiquitous genetic elements that play an essential role in multidrug tolerance and virulence *of bacteria*. So far, little is known about the TA systems in *Streptococcus suis*. In this study, the Xress-MNTss TA system, composed of the MNTss toxin in the periplasmic space and its interacting Xress antitoxin, was identified in *S. suis*. β-galactosidase activity and electrophoretic mobility shift assay (EMSA) revealed that Xress and the Xress-MNTss complex could bind directly to the Xress-MNTss promoter as well as downregulate streptomycin adenylyltransferase *ZY05719_RS04610*. Interestingly, the Xress deletion mutant was less pathogenic *in vivo* following a challenge in mice. Transmission electron microscopy and adhesion assays pointed to a significantly thinner capsule but greater biofilm-formation capacity in Δ*Xress* than in the wild-type strain. These results indicate that Xress-MNTss, a new type II TA system, plays an important role in antibiotic resistance and pathogenicity in *S. suis*.

## Introduction

Toxin-antitoxin (TA) systems are common in bacteria and archaea, in which they were initially discovered on plasmids (Ogura and Hiraga, 1983). An increasing number of TA systems have been identified in prokaryotic genomes (Pandey and Gerdes, 2005; Van Melderen and Saavedra De Bast, 2009), suggesting that they may play a critical role in the adaptation to stress (Gerdes et al., 2005; Wang and Wood, 2011; Yamaguchi and Inouye, 2011). TA systems are composed of a stable toxin capable of targeting essential cellular functions, such as DNA replication, mRNA stabilization, and peptidoglycan synthesis, plus an unstable antitoxin that counteracts the toxin’s activity (Harms et al., 2018). TA systems have been classified into seven types based on the nature and mode of action of the antitoxins (Wang et al., 2020). In type I TA system, the antitoxin is an antisense RNA that arrests the translation of the toxin by binding to the toxin mRNA (Gerdes and Wagner, 2007). In Type II TA system, antitoxin proteins neutralize toxins through direct protein-protein interactions (Leplae et al., 2011). In Type III TA system, RNA antitoxin neutralizes toxin proteins through direct protein-RNA interactions (Fineran et al., 2009). In Type IV TA system, the antitoxin counteracts the toxic effects of the toxin by interfering with the interaction of the toxin with its target (Masuda et al., 2012). In Type V TA system, the protein antitoxin inhibits the toxin by cleaving its mRNA (Wang et al., 2012). In VI type TA system, the binding of the antitoxin to the toxin triggers the degradation of the toxin by the protease (Markovski and Wickner, 2013). In type VII TA system, the antitoxin neutralizes the toxin protein through chemically modifying the toxin post-translationally (Wang et al., 2020).

In type *II TA* systems, the higA antitoxin of *Pseudomonas aeruginosa* binds to a palindromic sequence within the promoter region and represses transcription of TA operon (Guo et al., 2019). Stressful conditions trigger TA synthesis, whereby faster antitoxin degradation leads to more free toxin in the cell. Toxins of the TA locus affect bacterial functions in different ways to help cells adapt to stress or promote pathogenicity in the host (Gerdes et al., 2005; Lobato-Marquez et al., 2016). In addition, hok-sok and ccdAB, are responsible for the plasmids stabilization (Datta et al., 2017). SehAB contributes to bacterial virulence and RelBE promotes Vibrio cholerae colonization of the Intestine (De la Cruz et al., 2013; Wang et al., 2015). Specifically, SavRS can directly inhibit virulence genes, *hla* and *efb*, through antitoxin modulation (Wen et al., 2018). And MazF specifically cleaves a sequence, UACAU, which is abundant in the mRNA for pathogenic adhesive factor *sraP*. (Zhu et al., 2009). Yefm-YoeB and YbaJ-Hha modules promote bacterial colonization of mouse bladder (Norton and Mulvey, 2012). The above evidence indicates that TA systems are involved in drug resistance and pathogen-host interactions.

*Streptococcus suis* is an important and widely distributed pathogen that can cause severe infections in pigs and contribute to zoonotic diseases (Wertheim et al., 2009; Goyette-Desjardins et al., 2014). Symptoms of *S. suis* infection include arthritis, meningitis, endocarditis, and septicemia (Feng et al., 2014). *S. suis* can be classified into 33 reference serotypes based on capsular antigens, with *S. suis* serotype 2 (SS2) thought to be the most widespread (Gottschalk et al., 1993; Hill et al., 2005). Two serious *S. suis* outbreaks were recorded in China in 1998 and 2005 (Tang et al., 2006; Feng et al., 2010). Type II TA systems identified in *S. suis* include RelBE1, RelBE2, yefM-yoeB, and ParDE in strain SC84, as well as SezAT in strain 05ZYH33 (Yao X. et al., 2015; Zheng et al., 2015; Xu et al., 2018). Although the deletion of the yefM-yoeB had no effect on the virulence of SC84. SezAT promotes the maintenance of the SsPI-1 pathogenicity island in 05ZYH33. Studies on TA systems in *S. suis* have focused mostly on their toxic effects, and their role in the virulence of the strains needs to be further explored.

In this study, we identified a novel xenobiotic response element-minimal nucleotidyltransferase (Xre-MNT) family type II TA system in SS2 strain ZY05719. We report for the first time that a TA system such as Xress-MNTss mediates antibiotic resistance by controlling expression of drug resistance genes through auto-regulation. In addition, the lack of antitoxin leads to weakened pathogenicity in mice. These findings show that Xress-MNTss plays a vital role in the resistance of *S. suis* to streptomycin and virulence.

## Experimental Procedures

### Bacterial Strains, Plasmids, and Growth Conditions

The wild-type SS2 strain ZY05719 was isolated from a diseased pig during an outbreak in the Sichuan province of China. The bacterial strains and plasmids used in this study are listed in Table 1; the sequences of all primers are listed in Supplementary Table 1. *S. suis* strains were grown at 37°C in Todd–Hewitt broth (THB, Oxoid Cheshire, United Kingdom) or THB agar (THA). *Escherichia coli* strains were grown on Luria–Bertani (LB) agar plates or in LB broth at 37°C. For mutant selection, 100 μg/mL spectinomycin was added to *S. suis* medium. Ampicillin (100 μg/mL) was used to maintain plasmids pBADHisA and pBADHisA-pelB, and 50 μg/mL kanamycin or 150 μg/mL erythromycin was used to maintain the pTCV-*lac* plasmid.

**TABLE 1:** Bacterial strains and plasmids used in this study.

Bacterial strains/Plasmids	Description^a^	References
Strains		
ZY05719	Wild type	Collected in our laboratory
Δ*Xress-MNTss*	Xress-MNTss deletion mutant in ZY05719	This study
Δ*MNTss*	MNTss deletion mutant in ZY05719	This study
Δ*Xress*	Xress deletion mutant in ZY05719	This study
D-Xress	Antitoxin point mutant in ZY05719	This study
D-MNTss	Toxin point mutant in ZY05719	This study
Δcps	Capsular deletion strain in ZY05719	Collected in our laboratory
Top10	The expression host for pBADhisA and pBADhisA-pelB	Invitrogen
DH5α	Cloning host for maintaining the recombinant plasmids	Invitrogen
BL21 (DE3)	Host for expressing proteins	Invitrogen
Plasmids		
pBADHisA	Expression vector; Amp^*r*^	Invitrogen
pBADHisA-T2/T3	pBADHisA containing the T2/T3 gene; Amp^*r*^	This study
pBADHisA-MNTss	pBADHisA containing the MNTss gene; Amp^*r*^	This study
pBADHisA-T6	pBADHisA containing the T6 gene; Amp^*r*^	This study
pBADHisA-pelB	pBADHisA containing the PelB leader sequence; Amp^*r*^	This study
pBADHisA-pelB-T2/T3	pBADHisA-pelB containing the T2/T3 gene; Amp^*r*^	This study
pBADHisA-pelB-MNTss	pBADHisA-pelB containing the MNTss gene; Amp^*r*^	This study
pBADHisA-pelB-T6	pBADHisA-pelB containing the T6 gene; Amp^*r*^	This study
pBADHisA-pelB-Xress-MNTss	pBADHisA-pelB containing the Xress-MNTss gene; Amp^*r*^	This study
pTCV-Lac	Gram-positive bacteria-E. coli shuttle vector pTCV-lac; Kan^*r*^, Ery^*r*^	Poyart and Trieu-Cuot, 1997
pTCV-Lac-300	pTCV-Lac contains 300 bp of Xress-MNTss system promoter region; Kan^*r*^, Ery^*r*^	This study
pCold^TM^ II	Expression vector; Amp^*r*^	Invitrogen
pCold^TM^ II-Xress	pcold II containing the Xress gene; Amp^*r*^	This study
pCold^TM^ II-Xress-MNTss	pcold II containing the Xress-MNTss gene; Amp^*r*^	This study

### Growth Curve Determination

An overnight Top10 bacterial solution containing pBADHisA and pBADHisA-pelB plasmids was diluted 1:100 in fresh LB broth, supplemented with 100 μg/mL ampicillin (LB-ampicillin), and grown to OD_600_ of 0.2–0.3. Each culture was divided in two aliquots: one was supplemented with 0.2% L-arabinose and the other one not. A growth curve was constructed based on hourly OD_600_ measurements from at least three independent experiments. At the same time, after adding 0.2% L-arabinose, samples were diluted every 3 h and colony-forming units (CFU) were counted by spreading the serially diluted PBS in a 10-fold suspension of bacteria on LB agar plates for a 24 h of incubation period at 37°C.

### Bioinformatics Analysis

Nine putative type II TA systems in SS2 ZY05719 were predicted by TAfinder. DNAStar Lasergene 7^1^ and BLAST from NCBI^2^ were used to analyze DNA and amino acid sequences. The antitoxin and toxin three-dimensional (3D) structure was predicted using the SWISS-MODEL server, whereas the secondary structure was predicted using PHYRE2^2^. The promoter of Xress-MNTss was predicted using the SoftBerry website.

### Construction of Mutant Strains

Mutants were constructed *via* natural DNA transformation, with some modifications (Peterson et al., 2004; Zaccaria et al., 2014). The up and downstream sequences of the target gene were amplified by PCR with primer pairs, from the genomic DNA of strain ZY05719. The up and downstream sequences were fused with the sacB-spc cassette by overlap PCR. The linear fusion DNA fragment used for the mutants and synthetic peptide were added to the 100-ul bacteria [optical density at 600 nm (OD6_00_), 0.042]. To generate Δ*Xress-MNTss*, Δ*MNTss*, and Δ*Xress*, the composite samples were incubated at 37°C for 2 h under static conditions and then plated in THB containing spectinomycin. The *sacB* gene, which is sensitive to sucrose, was used as a negative control. Next, the fusion homologous fragment without any cassette was transferred to the primary positive mutant for the second transformation, after which the transformed bacteria were maintained on a THB plate containing 10% (w/v) sucrose. Construction of D-MNTss and D-Xress point mutant strains followed a similar protocol to that of deletion strains, except that the translation initiation codon ATG was mutated to CTG.

### Promoter Activity Assay

The Xress-MNTss promoter (300 bp) was amplified from ZY05719 genomic DNA. The PCR product was ligated in the pTCV-*lac* reporter plasmid to obtain pTCV-*lac*-300, which was then transferred to wild-type, deletion, and point mutant strains. The β-galactosidase activity assay was performed according to Miller’s method (Aviv and Gal-Mor, 2018) with some modifications. The overnight culture broth was diluted 1:100 with fresh THB and placed in a CO_2_ incubator at 37°C for cultivation. Upon reaching log phase (OD_600_ ∼ 0.6), 2 ml of bacterial cells culture was collected by centrifugation, washed twice with sterile phosphate-buffered saline (PBS), and resuspended in 200 μL pre-cooled Z-buffer containing 50 mM β-mercaptoethanol. Subsequently, 0.1% SDS and chloroform were added, the suspension was thoroughly mixed and placed in a 30°C water bath for 5 min; after which 100 μL *O*-nitrophenyl-β-D-galactopyranoside (4 mg/mL) was added, mixed well, and the reaction was allowed to proceed at 30°C until the solution was no longer yellow. At that point, 250 μL sodium carbonate was added to stop the reaction, the solution was centrifuged, and 250 μL of supernatant was aliquoted to a 96-well plate. Absorbance at 420 nm (A_420_) and 550 nm (A_550_) was recorded with a microplate reader, and β-galactosidase activity was calculated using the following formula:

Activity[MU]=[1,000×(A-4201.75×A)550]/[t(min)×v×OD]600,

where MU = Miller units; t = reaction time; and *v* = volume of culture assayed in milliliters. At least three independent cultures of each strain were assayed in each experiment.

### RNA Isolation, RT-PCR, and qRT-PCR

Whole-cell RNA from bacteria in log phase was extracted using TRIzol (TaKaRa) according to the manufacturer’s instructions. After removing any contaminating DNA with gDNA wiper, the extracted RNA served as template to synthesize cDNA using a HiScriptII first-strand cDNA synthesis kit (Vazyme). The QuantStudio 6 Flex RT-PCR system and ChamQ Universal SYBR qPCR master mix (Vazyme) were used to determine the concentration of selected transcripts. The housekeeping gene parC was used as an internal reference (Wu et al., 2014), and the 2^–Δ^ ^Δ^ ^*CT*^ method was employed to calculate the relative fold change. At least three replicates were performed for each sample (Livak and Schmittgen, 2001).

For the cotranscription test, total RNA was extracted using a bacterial RNA extraction kit and divided in two aliquots: one was reverse-transcribed into cDNA, and the other was not (negative control). Primers were designed to span *zy05719_RS04595*-*zy05719_RS04600*, *zy05719_RS04600*- *zy05719_RS04605*, and *zy05719_RS04605*- *zy05719_RS04610* (Supplexmentary Table 1). Negative controls and cDNA were used as templates for co-transcription analysis.

### Expression and Purification of Antitoxin and TA Protein Complex

The Xress coding sequence was amplified, cut with restriction enzymes, and ligated to pCold^TM^ II to generate the prokaryotic expression vector pCold^TM^ II-Xress. Separately, the antitoxin coding sequence was amplified, the stop codon was removed, the linker GGGGSGGGGSGGGGS was added to connect the antitoxin to the toxin coding sequence, and the construct was ligated in pCold^TM^ II to generate pCold^TM^ II-Xress-MNTss. The His-tag was placed at the N-termini of the protein. The pCold^TM^ II-Xress and pCold^TM^ II-Xress-MNTss plasmids were transformed into BL21(DE3) competent *E. coli*, which were cultured to OD_600_ ∼ 0.4–0.6. At that point, 0.5 mM isopropyl-D-thiogalactopyranoside was added and cells were cultured at 16°C for another 16 h. Next, the cells were harvested and sonicated in lysis buffer (20 mM Na_3_PO_4_⋅12H_2_O, 0.5 mM NaCl, 30 mM imidazole, pH 7.4), after which the antitoxin Xress and the Xress-MNTss protein complex were purified on a His-tag Ni-NTA affinity chromatography column. The protein was eluted with a step-wise gradient using imidazole concentrations ranging from 50 to 500 mM. The protein concentration was determined by performing a Bradford assay with bovine serum albumin as a standard.

### Electrophoretic Mobility Shift Assay

For EMSA, native polyacrylamide gel electrophoresis was performed by incubating the purified protein with Xress-MNTss promoter fragments probe. The latter (300 bp) were amplified by PCR and purified using a kit (TaKaRa). The negative-control probe was amplified from 16S rRNA. The purified protein and DNA probe were added to the binding buffer (10 mM Tris-base, 50 mM KCl, 5 mM MgCl_2_, 1 mM dithiothreitol, 0.05% Nonidet P-40, 2.5% glycerol, pH 7.5), incubated at 37°C for 30 min, and then subjected to 6% native polyacrylamide gel electrophoresis in 0.5 × TBE buffer (44.5 mM Tris-base, 44.5 mM boric acid, 1 mM EDTA, pH 7.5) at 200 V for 45 min. The gel was stained in 0.5 × TBE containing ethidium bromide for 20 min and images were taken.

### Antimicrobial Susceptibility Assays

The MICs of antibiotics against *S. suis* were determined according to the Clinical and Laboratory Standards Institute Guidelines (Humphries et al., 2018). The strains were diluted 1,000-fold in THB, and 180 μL of the inoculated culture was added to the first well of a 96-well microtiter plate and 100 μL to the other wells. Next, 20 μL antibiotics was added to the first well, mixed, and 100 μL of the mixture was added to the following well. The procedure was repeated until the 10th well. The 11th well served as the positive control and the 12th well as the negative control. The 96-well plate was then incubated at 37°C for 20 h, and the results were recorded. Each experiment was repeated independently three times.

### Mouse Infection Assay

To assess the virulence of the deletion strain *in vivo*, we randomly divided BALB/c mice into five groups of 10 mice each, and challenged them with 5 × 10^8^ colony-forming units (CFU)/mouse. The control group was challenged with PBS. Clinical symptoms and survival of the mice were monitored for 7 days. Additionally, a bacterial load assay was performed to evaluate the proliferation capacity *in vivo*. Each group consisted of six mice, and the intraperitoneal injection dose used was 3 × 10^8^ CFU/mouse of ZY05719 and Δ*Xress*. At 6 h postinfection, the mice were anesthetized with isoflurane and euthanized by CO_2_. Blood, brains, livers, and spleens were harvested, weighed, and homogenized in PBS. Bacteria were isolated from these homogenates and blood by plating serial 10-fold dilutions on a THB-agar medium to enumerate CFU. Animal experiments were conducted at the Animal Center Laboratory of Nanjing Agricultural University and were approved by the Jiangsu Provincial Laboratory Animal Monitoring Committee.

### Biofilm Assay

Biofilm formation was analyzed by staining with 0.1% crystal violet in a 96-well plate. Briefly, bacteria were grown to OD_600_ = 0.6, diluted 1:100, inoculated into a 96-well plate, and incubated at 37°C for 3 days. THB served as a control. The medium was discarded and the cells were gently washed twice with PBS to remove any unattached bacteria. After fixing with methanol for 30 min, the fixation solution was discarded, the samples were dried at room temperature, and the biofilm was stained with 0.1% crystal violet for 30 min. The samples were washed with tap water, dried, 33% acetic acid was added, the mixture was placed on a shaker at 80 rpm/min for 30 min to release crystal violet, and absorbance was measured at 600 nm. Ten independent cultures were used for each strain.

### Adhesion Test With Hep-2 Epithelial Cells

Adhesion experiments were performed *in vitro* according to established methods. Briefly, Hep-2 human laryngeal epithelial cells were cultured in Dulbecco’s modified Eagle medium (DMEM) containing 10% fetal bovine serum until they reached a monolayer in 24-well cell plates (5 × 10^5^ cells/well). The bacteria were cultured to log phase, washed with DMEM, and diluted to a density of 5 × 10^7^ CFU/mL. Each cell plate well was infected with 1 mL of bacterial solution, centrifuged at 800 × *g* for 15 min, and incubated at 37°C for 2 h. The cells were washed five times with sterile PBS to remove floating bacteria, treated with 100 μL of 0.25% trypsin-EDTA at 37°C for 10 min, and rinsed with 900 μL sterile deionized water to release any bacteria adhering to the cells. Finally, the bacteria were diluted and counted on a THB plate. The results were expressed in terms of relative adhesion frequency, compared with the adhesion frequency of the wild-type (set to 100%). Data were obtained from at least three independent experiments.

### Dot Blot Assay

To quantify the level of capsule production, we performed a dot blot assay. Briefly, 5 μL of bacteria, twofold serially diluted in PBS, were spotted onto a nitrocellulose membrane and fixed with 70% ethanol for 5 min. After air-drying, the membranes were blocked with blocking solution (5% w/v skim milk in PBS containing 0.05% Tween 20) for 2 h. Anti-SS2 polyclonal antibody (1:500 dilution) was applied to probe the nitrocellulose membrane spotted with bacteria. After washing with PBS-Tween 20 buffer, the membrane was incubated with horseradish peroxidase-conjugated anti-rabbit secondary antibody (1:2,000 dilution, Boster). The signal was detected using the Tanon High-sig ECL western blotting kit. The average gray value was quantified using ImageJ software^3^. The experiment was repeated three times, and the ZY05719 capsule-deletion strain (Δ*CPS*) was used as a control.

### Transmission Electron Microscopy

*Streptococcus suis* morphology was observed using a transmission electron microscope. Briefly, bacteria were grown to mid-exponential phase, centrifuged at 5,000 × *g* for 10 min, and then fixed with 2.5% glutaraldehyde for at least 2 h. The samples were dehydrated in propylene oxide for 10 min and visualized using a Hitachi H-7650 apparatus according to the manufacturer’s instructions. This experiment was performed independently three times.

### Statistical Analyses

All experiments were repeated at least three times. GraphPad Prism version 8 was used for analysis and plotting. Statistical significance was set to *P* < 0.05, and an unpaired two-tailed Student’s *t*-test or log-rank (Mantel-Cox) test was applied to analyze the data.

## Results

### Bioinformatics Analysis and Identification of Xress-MNTss in SS2 Strain ZY05719

Nine putative type II TA systems in SS2 strain ZY05719 were predicted using TAfinder (Table 2), an online tool in TADB (Shao et al., 2011). The localization of TA systems in the complete genome of ZY05719 and related sequence information are shown in Supplementary Figure 1. Based on a comparative analysis, TA1, TA4, TA7, TA8, and TA9 were found to be homologous to type II TA systems RelBE1 (SC84), SezAT (05ZYH33), RelBE2 (SC84), yefM-yoeB (SC84), and ParDE (SC84), respectively (Yao X. et al., 2015; Zheng et al., 2015; Xu et al., 2018). Instead, TA2, TA3, Xress-MNTss, and TA6 have not been characterized yet.

**TABLE 2:** Nine putative type II toxin-antitoxin (TA) systems in *S. suis* ZY05719.

TA-NO.	Toxin	Antitoxin	TA domain pair^1^
TA1	*ZY05719_RS03015*	*ZY05719_RS03010*	RHH-RelE
TA2	*ZY05719_RS04255*	*ZY05719_RS04250*	-
TA3	*ZY05719_RS04255*	*ZY05719_RS04260*	RHH-RelE
TA4	*ZY05719_RS04510*	*ZY05719_RS04515*	-
TA5	*ZY05719_RS04600*	*ZY05719_RS04595*	Xre-MNT
TA6	*ZY05719_RS05490*	*ZY05719_RS05495*	-
TA7	*ZY05719_RS07085*	*ZY05719_RS07090*	RHH-RelE
TA8	*ZY05719_RS09470*	*ZY05719_RS09475*	PHD-RelE
TA9	*ZY05719_RS09485*	*ZY05719_RS09490*	RHH-RelE

*^1^TA domain pair represents the protein domain pair of toxin and cognate antitoxin. -, it means no TA domain pair.*

**FIGURE 1:**
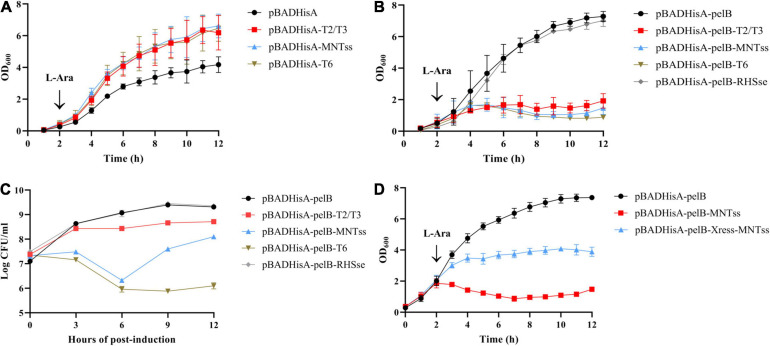
Effect of toxin and antitoxin induction on growth of *E. coli* Top10 cells. Toxins were linked to pBADHisA and pBADHisA-pelB plasmids and transformed into *E. coli* Top10 cells. Cytoplasmic toxicity in cells expressing the toxin on pBADHisA **(A)** with 0.2% L-arabinose. Periplasmic toxicity in cells expressing the toxin on pBADHisA-pelB **(B)** with L-arabinose. **(C)** Viability of strains expressing T2/T3, MNTss, T6, and RHSse. To quantitate CFU per milliliter, cells were diluted and plated on LB agar plus 100 ug of ampicillin/ml at the times indicated. Growth of *E. coli* Top10 cells containing the pBADHisA-pelB-Xress-MNTss plasmid with 0.2% L-arabinose **(D)** to determine the neutralization effect of the antitoxin. Culture growth was monitored by measuring OD_600_ every hour. Growth curves represent at least three independent experiments.

Next, we identified the putative TA systems. Using the pBADHisA plasmid, we found that the toxins T2/T3, MNTss, and T6 elicited no toxic effect in the cytoplasm, with addition of the inducer L-arabinose (Figure 1A). Thus, we chose plasmid pBADHisA-pelB to induce toxin secretion in the periplasmic space. The periplasmic localization (pBADHisA-pelB) was achieved by fusion to the PelB leader sequence (Lei et al., 1987). In order to rule out the possibility of protein accumulation in the periplasm and lead to growth arrest, we added a negative control (pBADHisA-pelB-RHSse). Growth of the resulting *E. coli* strain slowed significantly when L-arabinose was added, indicating a bactericidal effect (Figure 1B). The accumulation of RHSse (QRR36965.1) protein in the periplasm did not lead to growth inhibition, indicating that the growth inhibition caused by T2/T3, MNTss, and T6 was due to toxic effects. And T2/T3 showed bacteriostatic effect, while MNTss and T6 showed significant bactericidal effect (Figure 1C). These results demonstrated that the toxins T2/T3, MNTss, and T6 exerted a toxic effect in the periplasmic space rather than the cytoplasm. To identify the antitoxin of MNTss, we ligated the antitoxin and toxin coding sequences in pBADHisA-pelB, and assessed bacterial growth to determine if the antitoxin neutralized the toxin. As shown in Figure 1D, *E. coli* containing toxin-only pBADHisA-pelB-MNTss was significantly inhibited, whereas that harboring pBADHisA-pelB-Xress-MNTss could largely neutralize the toxin, indicating that MNTss and Xress formed an active TA system.

Further, the 3D structure of the antitoxin Xress (Supplementary Figure 2A) and toxin MNTss (Supplementary Figure 3A) was predicted using the SWISS-MODEL protein homology-modeling server^4^. Xress was predicted to belong to the Xre family (Supplementary Figure 2B) of transcriptional regulators harboring a helix-turn-helix domain. MNTss was predicted to belong to the MNT family (Supplementary Figure 3B) and contain a nucleotidyltransferase domain similar to the kanamycin nucleotidyltransferase of *Staphylococcus aureus*. So we named this TA system Xress-MNTss.

### Identification of Xress and Xress-MNTss Regulation

**FIGURE 2:**
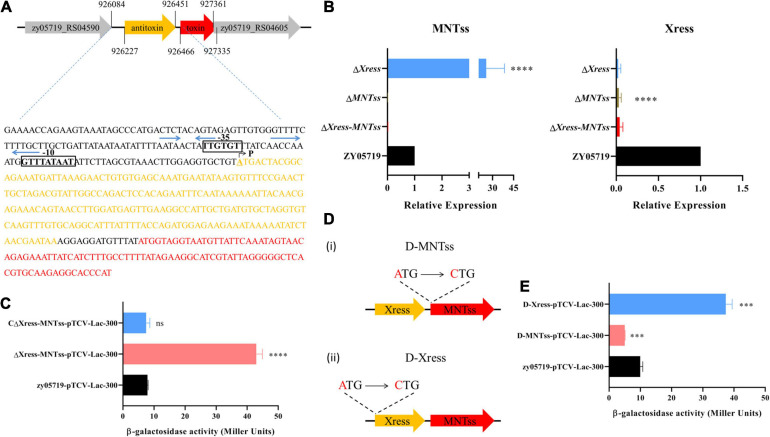
Evaluation of relative expression and promoter activity *in vivo*. **(A)** Predicted Xress-MNTss promoter. Gray areas comprise the -35 box and -10 box regions, blue arrows represent the predicted palindrome sequences. P indicates the transcriptional start site (TSS). **(B)** Expression levels of MNTss and Xress in ZY05719, Δ*Xress-MNTss*, Δ*MNTss*, and Δ*Xress* strains as measured by qRT-PCR. The relative expression levels represented the mean ± SD of three biological repeats. **(C)** The pTCV-*lac* reporter plasmid (pTCV-*lac*-300), containing the Xress-MNTss promoter sequence, was transferred to Δ*Xress-MNTss*,CΔ*Xress-MNTss* and ZY05719 to determine β-galactosidase activity. **(D)** (i) Toxin point mutant D-MNTss was constructed by mutating the start codon ATG of MNTss to CTG; (ii) antitoxin point mutant D-Xress was constructed by mutating the start codon ATG of Xress to CTG. **(E)** Promoter assay using pTCV-*lac*-300 integrated in the D-MNTss and D-Xress host. The data are shown as the means and standard deviations of the results from three independent experiments performed in triplicate. Unpaired two-tailed Student’s *t*-test: ns, *P* > 0.05; ^∗∗∗^*P* < 0.001; ^****^P < 0.0001.

Since studies have reported that antitoxin has a regulatory effect (Guo et al., 2019), and the previous analysis found that Xress has the potential to regulate genes. So we analyze the gene sequence structure of the Xress-MNTss system, the SoftBerry website^5^ was used to predict the Xress-MNTss promoter (Figure 2A). Several pairs of palindromic sequences were found in the Xress-MNTss promoter using an online prediction website^6^. The antitoxin or TA complex of a typical type II TA system can bind to its own promoter to ensure auto-regulation. To determine whether this was the case of Xress-MNTss, we constructed theΔ*Xress-MNTss* and CΔ*Xress-MNTss* strains, as well as inserted the Xress-MNTss promoter in the pTCV-*lac* plasmid. An *in vivo* promoter activity assay revealed significantly more β-galactosidase activity in the Δ*Xress-MNTss* strain compared to the wild-type and CΔ*Xress-MNTss*, suggesting that the Xress-MNTss system was capable of inhibiting its own promoter (Figure 2C).

To directly determine whether the antitoxin regulated the Xress-MNTss promoter *in vivo*, Δ*Xress* and Δ*MNTss* were constructed (Supplementary Figure 4), toxin and antitoxin expression was measured in the Δ*MNTss*, Δ*Xress* and Δ*Xress-MNTss* mutants. Toxin expression was 25-fold higher in the Δ*Xress* mutant than in the wild-type, whereas the antitoxin was significantly downregulated (Figure 2B). Because these results suggested that the antitoxin Xress was likely to negatively regulate the Xress-MNTss promoter, toxin and antitoxin point mutants were constructed (Figure 2D). Transcription of toxin in the antitoxin point mutant was significantly upregulated, and transcription of antitoxin was significantly downregulated in toxin point mutation (Supplementary Figure 5). The pTCV-*lac* plasmid was transformed into the point mutant strains and Δ*Xress* to analyze promoter activity *in vivo*. As expected, promoter activity was fourfold higher in the antitoxin point mutant (Figure 2E) and sixfold higher in the Δ*Xress* (Supplementary Figure 6). This finding indicated that the antitoxin exerted a negative regulatory effect on the Xress-MNTss system.

**FIGURE 3:**
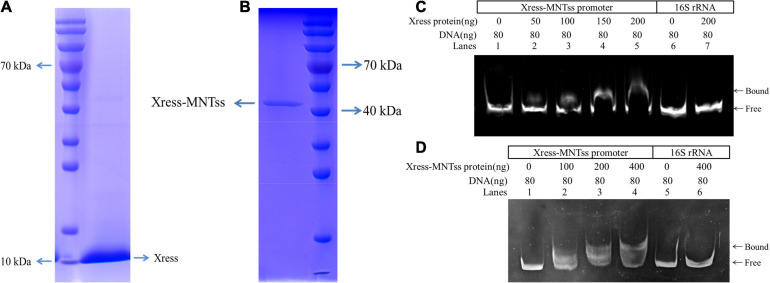
EMSA analysis *in vitro*. **(A)** Purification of Xress-His protein from pCold^TM^ II-Xress-containing BL21(DE3) *E. coli*. **(B)** Purification of Xress-MNTss-His protein from pCold^TM^ II-Xress-MNTss containing BL21(DE3) *E. coli*. **(C)** EMSA result showing binding of the antitoxin Xress to the Xress-MNTss promoter. The purified Xress protein was added to each reaction mixture at different concentrations. DNA probes containing the Xress-MNTss operon promoter region were used at 80 ng per reaction mixture (lanes 1–5). And fragments amplified from 16S rRNA served as a negative control (lanes 6–7). **(D)** TA Xress-MNTss complex bound to the Xress-MNTss promoter. The Xress-MNTss promoter region could be shifted by the TA complex in EMSA. DNA probes containing the Xress-MNTss operon promoter region were used at 80 ng per reaction mixture (lanes 1–4). And fragments amplified from 16S rRNA served as a negative control (lanes 5–6).

And in order to assess direct binding of Xress-MNTss to its own promoter, an electrophoretic mobility shift assay (EMSA) was conducted with purified antitoxin protein (Figure 3A) and TA complex (Figure 3B). The antitoxin Xress and TA Xress-MNTss complex could retard the mobility of the Xress-MNTss promoter in a dose-dependent manner (Figures 3C,D), revealing that the antitoxin and TA complex bound directly to the Xress-MNTss promoter to regulate the Xress-MNTss system.

### The Xress-MNTss System Affects Streptomycin Resistance

**FIGURE 4:**
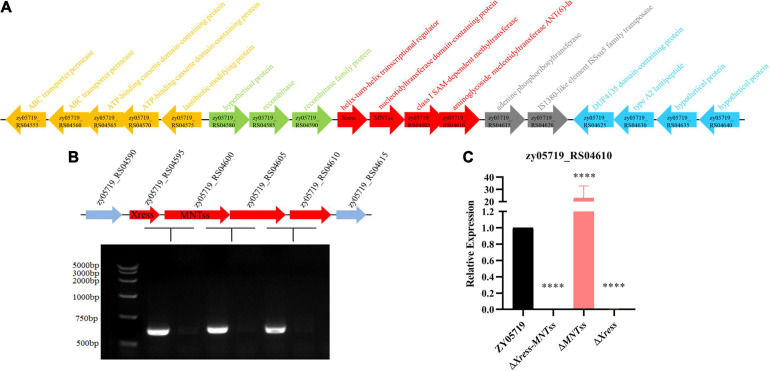
Xress-MNTss regulates the *zy05719_RS05610* gene. **(A)** Putative genomic island genes annotation. **(B)** *ZY05719_RS04595*, *zy05719_RS04600*, *zy05719_RS04605*, and *zy05719_RS04610* formed an operon, as determined by RT-PCR. An RNA sample without reverse transcription served as a negative control. **(C)** Expression of *zy05719_RS04610* in ZY05719, Δ*Xress-MNTss*, Δ*MNTss*, and Δ*Xress* strains as measured by qRT-PCR. Values are shown as the means plus standard deviations (error bars) from at least three independent experiments. Unpaired two-tailed Student’s *t*-test: *P* < 0.0001.

Further use the island viewer website^7^, the Xress-MNTss system was found to be located on a putative genomic island (Figure 4A). Gene structure analysis revealed a streptomycin resistance gene (*zy05719_RS04610*) downstream of the Xress-MNTss system, and prediction by the BPROM suite in SoftBerry showed that *zy05719_RS04595–zy05719_RS04610* formed a single operon and shared the same promoter (Figure 4B). *ZY05719_RS04610* is homologous to streptomycin adenylyltransferase (*SSUSC84_0863*) in *S. suis* SC84. Given that the antitoxin Xress and Xress-MNTss complex bound to the Xress-MNTss promoter and had a negative auto-regulatory effect, the Xress-MNTss system likely regulated the expression of *zy05179_RS04610*, too. Indeed, *zy05719_RS04610* was significantly downregulated in Δ*Xress-MNTss* and Δ*Xress*, but significantly upregulated in Δ*MNTss* (Figure 4C), reflecting the higher sensitivity to streptomycin in the former two and resistance in the latter (Table 3). Hence, *zy05719_RS04610* might mediate resistance to streptomycin in ZY05719. However, the regulation mechanism of streptomycin resistance needs to be further explored.

**TABLE 3:** The MICs of strain to Streptomycin.

Antimicrobial	Strain			
	ZY05719	Δ*Xress-MNTss*	Δ*MNTss*	Δ*Xress*
Streptomycin	256 μg/ml	8 μg/ml	>512 μg/ml	8 μg/ml

### The Xress-MNTss System Is Involved in Biofilm Formation and Required for Full Virulence

**FIGURE 5:**
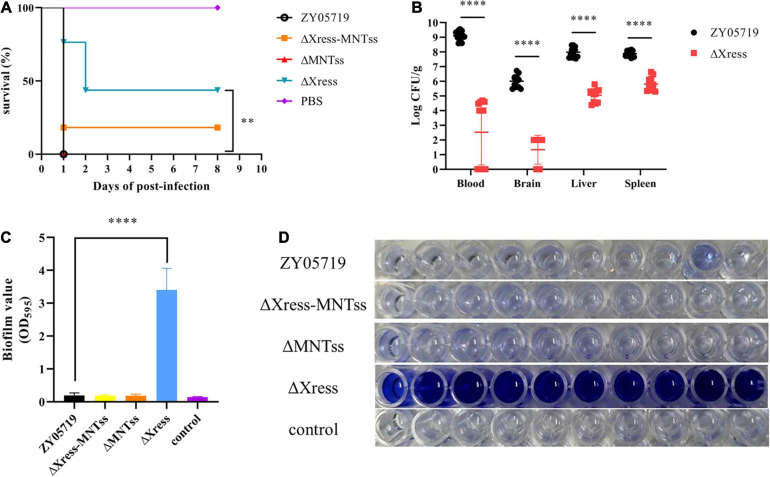
Xress-MNTss contributes to *S. suis* virulence and affects biofilm formation. **(A)** Survival curves of 5-week-old BALB/c mice infected with wild-type or mutant strains at 5 × 10^8^ CFU/mouse. The control group received only PBS. Ten mice from each group were monitored over a 7-day period. Log-rank (Mantel-Cox) test to determine differences in survival between groups: ^∗∗^*P* < 0.01. **(B)** Infected mice were euthanized 6 h after infection to determine the bacteria burden in the blood, brain, liver, and spleen. Biofilm formation in panel **(C,D)** 200 μl culture medium was inoculated in each well of 96-well plate. Unpaired two-tailed Student’s *t*-test: ^****^P < 0.0001.

Because Xress has the ability to regulate target genes, we wondered whether Xress also regulates other genes to cause corresponding phenotypic changes. And given that the TA system has been reported to participate in biofilm formation, this characteristic was investigated. Biofilm formation was significantly increased in the Δ*Xress* strain compared to that in the wild-type (Figures 5C,D), confirming the involvement of the Xress-MNTss system.

**FIGURE 6:**
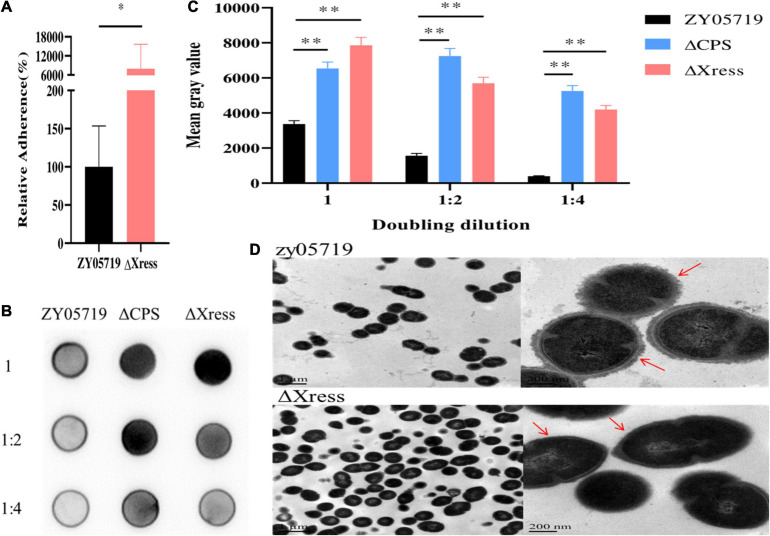
Capsule synthesis is reduced in the Δ*Xress* mutant. **(A)** Adhesion of Δ*Xress* to Hep-2 epithelial cells. The adhesion rate of the ZY05719 strain was significantly lower than that of the Δ*Xress* strain. Values represent the average of three independent replicates. Two-tailed unpaired Student’s *t*-test: ^∗^*P* < 0.05. **(B)** Dot-blot membrane showing capsule productions in ZY05719, Δ*CPS* (control), and Δ*Xress*. Dilutions of cells were made in PBS and spotted onto nitrocellulose membranes; the cells were then fixed and the membranes were probed with the absorbed antibody. **(C)** The quantitative analysis of Dot-blot result. Two-tailed unpaired Student’s *t*-test: ^∗∗^*P* < 0.01. **(D)** Transmission electron micrographs showing capsule thickness in ZY05719 and Δ*Xress*; scale bars, magnification level. The scale bars indicate the magnification size.

Based on reports of the involvement of the TA system in pathogenicity, BALB/c mice were used to explore whether the Xress-MNTss system contributed to bacterial virulence. Mice infected with ZY05719, Δ*Xress-MNTss*, and Δ*MNTss* strains showed clinical symptoms and mortality rates of 100%, 90%, and 100%, respectively. In contrast, survival was significantly increased in mice infected with Δ*Xress* (Figure 5A). And the bacterial abundances of Δ*Xress* in the blood, brain, liver, and spleen were significantly lower than that of ZY05719 (Figure 5B). No significant growth difference was observed between wild-type and mutant strains when cultured in THB (Supplementary Figure 7). These results showed that the Xress-MNTss system contributed to pathogenicity of *S. suis* ZY05719. Furthermore, Δ*Xress* adhered significantly better to Hep-2 cells than the wild-type (Figure 6A). Previously, loss of the *S. suis* capsule was shown to increase adhesion to epithelial cells. Here, dot blot analysis was used to verify loss of the capsule in Δ*Xress*. Compared to the wild-type strain, the dot-blot signal was significantly higher in Δ*Xress*, and was comparable to the Δ*CPS* control (Figures 6B,C). Transmission electron microscopy analysis of wild-type and Δ*Xress* cells showed significantly decreased capsule thickness in the latter (Figure 6D). Taken together, these results suggested that the Xress-MNTss system participated in biofilm formation and capsule synthesis.

## Discussion

Toxin-antitoxin systems exert various biological functions. The present study identified a new type II TA system in *S. suis*, and explored its role in drug tolerance and virulence. The results can be summarized as follows: (i) the toxin MNTss was identified as part of a periplasmic type II TA system together with the antitoxin Xress; (ii) Xress and the Xress-MNTss complex bind directly to the Xress-MNTss promoter to achieve negative regulation; (iii) Xress-MNTss regulates the streptomycin resistance gene through auto-regulation; (iv) Xress-MNTss affects biofilm formation and capsule synthesis in *S. suis* ZY05719. These results reveal the important role of Xress-MNTss in drug resistance and virulence in *S. suis*.

Several type II TA systems have been reported, including MazEF, HigBA, RelBE, MqsRA, HipBA, YefM-YoeB, VapBC, ω-ε-ζ, PezTA, Phd/Doc, and ParDE (Aizenman et al., 1996; Camacho et al., 2002; Meinhart et al., 2003; Kamada and Hanaoka, 2005; Takagi et al., 2005; Khoo et al., 2007; Brown et al., 2009; Christensen-Dalsgaard et al., 2010; Chan et al., 2012, 2014, 2018; Lioy et al., 2012; Wen et al., 2014; Kedzierska and Hayes, 2016). In this study, the toxin MNTss inhibited *E. coli* growth when located in the periplasmic space but not in the cytoplasm (Figure 1). This is not the same as the previously reported toxins, because those toxins exert toxic effects in the cytoplasm. The reason why the toxins exert toxicity in the periplasm but not in the cytoplasm may be that the toxins cannot be folded correctly in the cytoplasm. Because the periplasm of gram-negative bacteria can provide an environment for protein oxidation, folding and quality control (Miller and Salama, 2018). Although different combinations of toxins and Xre family antitoxins have been identified, such as RES-Xre, XRE-DUF397, tad-ata, MbcTA, and HicAB (Dziewit et al., 2007; Li et al., 2016; Santamaria et al., 2018; Freire et al., 2019; Skjerning et al., 2019), the Xre-MNT pair has not been reported. Based on bioinformatics analysis, MNT in the HEPN-MNT module was predicted to act as a toxin; however, a genome-scale screening of toxic proteins concluded that it was an antitoxin (Makarova et al., 2009; Kimelman et al., 2012; Yao J. et al., 2015). In this study, we confirm that MNT is indeed an active toxin with a strong bactericidal effect on *E. coli* and, consequently, Xre-MNT represents a novel TA family.

In type II TA systems, antitoxins bind to the promoter region and repress operon transcription (Yang and Walsh, 2017). The analysis of β-galactosidase activity and EMSA found that Xress also has the function of binding to the promoter and inhibiting the transcription of the operon. In *S. aureus*, the antitoxin SavR can bind to the palindromic sequence in the promoter region, and the toxin SavS forms a complex with SavR to enhance promoter binding, thereby enhancing auto-regulation (Wen et al., 2018). In contrast, in *P. aeruginosa*, toxin HigB forms a complex with the antitoxin HigA to block this process (Guo et al., 2019). We found that the Xress-MNTss promoter region also contains palindrome sequences. Whether Xress also binds to palindrome sequences needs to be further confirmed. The Xress-MNTss protein complex, like SavRS, can also bind to the promoter to achieve negative regulation. Auto-regulation has been documented in other TA systems, such as MazEF in *E. coli*, MqsRA in *Xylella fastidiosa*, CcdAB in *E. coli* F plasmid, and AbiE in *Streptococcus agalactiae* (Merfa et al., 2016; Nikolic et al., 2018; Beck et al., 2020). This mode of TA regulation allows for the rapid release of high levels of toxins after bacteria degrade unstable antitoxins under stress conditions.

Toxin-antitoxin systems were initially related to the maintenance of plasmids, and plasmids often harbor genes that benefit bacteria by providing protection against antibiotics (Ogura and Hiraga, 1983; Gerdes et al., 1986). In addition, the TA system stabilizes genome islands, as in the case of the sgiAT system encoded by the multidrug resistance *Salmonella* Genomic Island 1 (Huguet et al., 2016). The mosAT TA system helps maintain the integrity of the SXT integrative and conjugative element that mediates resistance to multiple antibiotics in clinical isolates of *Vibrio cholerae* (Wozniak and Waldor, 2009). *Mycobacterium tuberculosis* grown at 10 × the minimal inhibitory concentration (MIC) of vancomycin revealed the induction of two TA systems, confirming their role against antibiotics (Provvedi et al., 2009). There are many pathways in which the TA system contributes to multidrug tolerance, such as affecting the formation of biofilms, promoting the maintenance of ICE, and forming persisters. Our study complements the pathway the TA system contributes to antibiotic resistance, that is, through self-regulation to regulate downstream resistance genes. Although the regulation of *zy05719_RS04610* by Xress-MNTss was inconsistent with the displayed phenotype, possibly as a result of overlapping regulatory networks, the MICs and gene transcription levels were consistent. This shows that *zy05719_RS04610* may mediate resistance of ZY05719 to streptomycin.

TA systems are more abundant in pathogens associated with severe or chronic infections compared to those that are non-pathogenic or cause only mild diseases (Pandey and Gerdes, 2005). Bacterial loads and pathological damage in guinea pig tissues were significantly lower with the *M. tuberculosis* MazF triple mutant than with the wild-type (Tiwari et al., 2015). Our results show that the absence of Xress weakened the virulence of the strain and affected the formation of biofilms. Although TA systems affect the production of biofilms (Wen et al., 2014; Wood and Wood, 2016), the mechanism underlying the increase in Δ*Xress* biofilm formation requires further study. Deletion of Δ*Xress* significantly increased adhesion to Hep-2 cells. Greater adhesion of *S. suis* to epithelial cells has been often associated with capsule loss (Lalonde et al., 2000; Benga et al., 2004). Dot-blot analysis and transmission electron microscopy also confirmed that the capsule of Δ*Xress* was significantly thinner. As quantitative real-time PCR (qRT-PCR) revealed no significant decrease in transcription of the capsular gene cluster (data not shown), other regulatory mechanisms seem to be affected. The further deletion of MNTss encoding gene in the ΔXress strain could not restore the change caused by the deletion of Xress encoding gene in the antibiotic resistance tests, suggested that this phenotype was not related with the upregulation of MNTss in ΔXress strain. However, compared with Δ*Xress-MNTss* and Δ*MNTss*, both of which remained strongly virulent, the low virulence of Δ*Xress* could be due to a thinner capsule caused by toxins, rather than deletion of the antitoxin Xress. While such an effect of MNT on bacterial virulence has not been documented in other TA models, it could point to a previously unknown mechanism. The further experiments need to be performed to confirm the potential effect on the biofilm formation and biosynthesis caused by the upregulation of MNTss in ΔXress strain.

**FIGURE 7:**
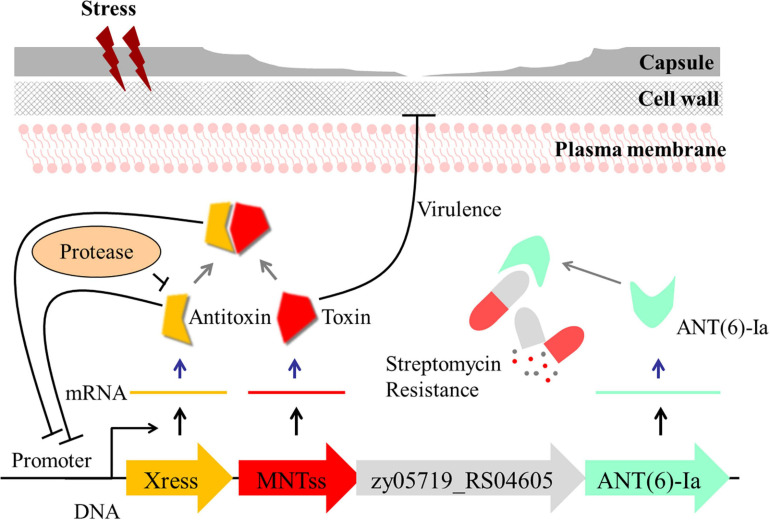
Model illustrating the proposed regulation of drug resistance and role of Xress-MNTss in *S. suis* virulence. Under non-stressful conditions, Xress binds directly to MNTss to neutralize the toxicity of MNTss, while Xress and the Xress-MNTss complex bind directly to the promoter region to achieve auto-regulation. Under stressful conditions, the antitoxin is degraded by proteases, MNTss is released, the inhibitory effect of the toxin on *zy05719_RS04605* and *zy05719_RS04610* is removed. At the same time, MNTss accumulates in the cytoplasm, affecting capsule thickness and biofilm formation with consequent decrease in ZY05719 virulence.

Based on these findings, we propose a model, whereby Xress-MNTss regulates antibiotic resistance and participates in *S. suis* virulence (Figure 7). The Xress and Xress-MNTss complex bind to the promoter region to inhibit transcription of Xress, MNTss, *zy05719_RS04605*, and *zy05719_RS04610*. Under stressful conditions, Xress is rapidly degraded by proteases. In the absence of Xress inhibition or the Xress-MNTss complex, MNTss is quickly released, and the de-inhibition of the operon will affect the expression of the downstream genes, which may cause the significant changes of streptomycin resistance. At the same time, the accumulation of MNTss negatively affects the capsule while promoting biofilm formation, two traits that are closely related to bacterial virulence. Future experiments should focus on identifying other pathways that regulate streptomycin resistance genes, other toxin targets, and the reasons for the reduced capsule synthesis.

## Data Availability Statement

The original contributions presented in the study are included in the article/Supplementary Material, further inquiries can be directed to the corresponding author/s.

## Ethics Statement

The animal study was reviewed and approved by Jiangsu Provincial Laboratory Animal Monitoring Committee.

## Author Contributions

HY and ZP conceived the idea, and designed and supervised the experiments. QG performed *S. suis* microbiology experiments. QG and PH performed mice experiments. DW performed growth experiments. JM and XZ performed data analyses. YZhu, YZha, and QB performed protein purification. HY, ZP, and QG wrote the manuscript. All authors contributed to the article and approved the submitted version.

## Conflict of Interest

The authors declare that the research was conducted in the absence of any commercial or financial relationships that could be construed as a potential conflict of interest.

## Publisher’s Note

All claims expressed in this article are solely those of the authors and do not necessarily represent those of their affiliated organizations, or those of the publisher, the editors and the reviewers. Any product that may be evaluated in this article, or claim that may be made by its manufacturer, is not guaranteed or endorsed by the publisher.

## Footnotes

1
https://blast.ncbi.nlm.nih.gov/Blast.cgi

2
http://www.sbg.bio.ic.ac.uk/phyre2/

3
https://imagej.nih.gov/ij/

4
http://swissmodel.expasy.org/

5
http://linuxl.softberry.com/

6
http://emboss.bioinformatics.nl/cgi-bin/emboss/palindrome

7
http://www.pathogenomics.sfu.ca/islandviewer/browse/
