# Supplementary material for: An Auto-Regulating Type II Toxin-Antitoxin System Modulates Drug Resistance and Virulence in Streptococcus suis
Source: Front Microbiol. 2021 Aug 12;12:671706. doi: 10.3389/fmicb.2021.671706 (PMC8406773; doi:10.3389/fmicb.2021.671706)
Supplement: Supplementary file 1 [file Data_Sheet_1.DOCX]

Supplementary Material

## Supplementary Figures

**
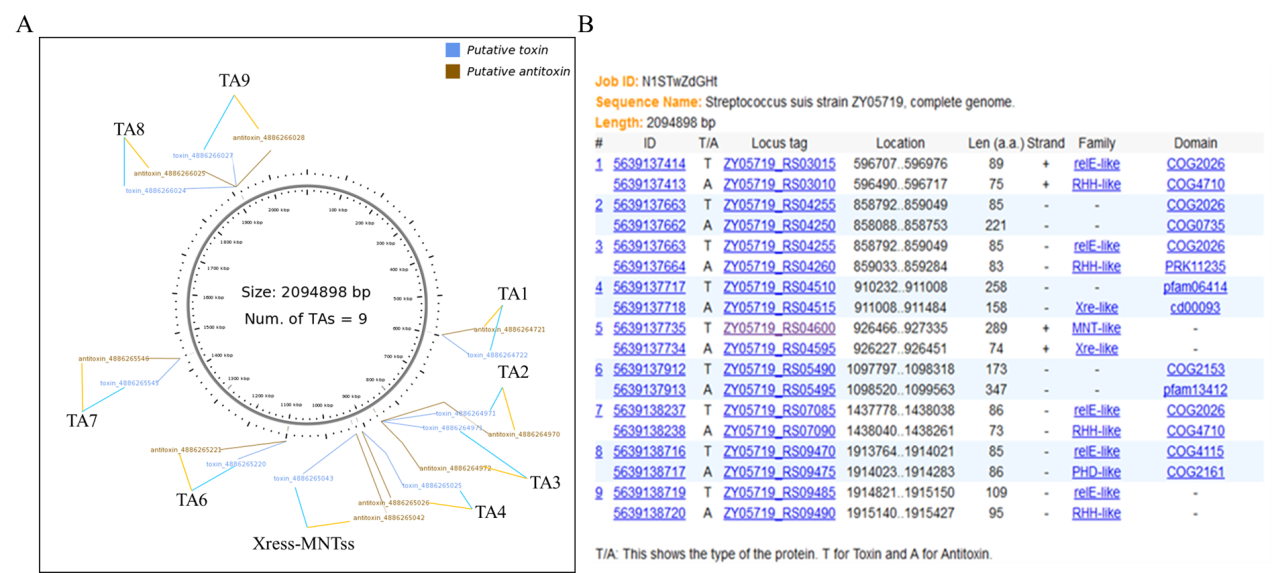
**

**Supplementary Figure S1. Putative toxin-antitoxin systems in ZY05719 chromosome.** (A) The position of the toxin-antitoxin systems on the circular genome map, and TA1-TA4, Xress-MNTss, TA6-TA9 represent the corresponding toxin-antitoxin system. (B) ID numbers of each toxin or antitoxin and related information about the gene sequence.


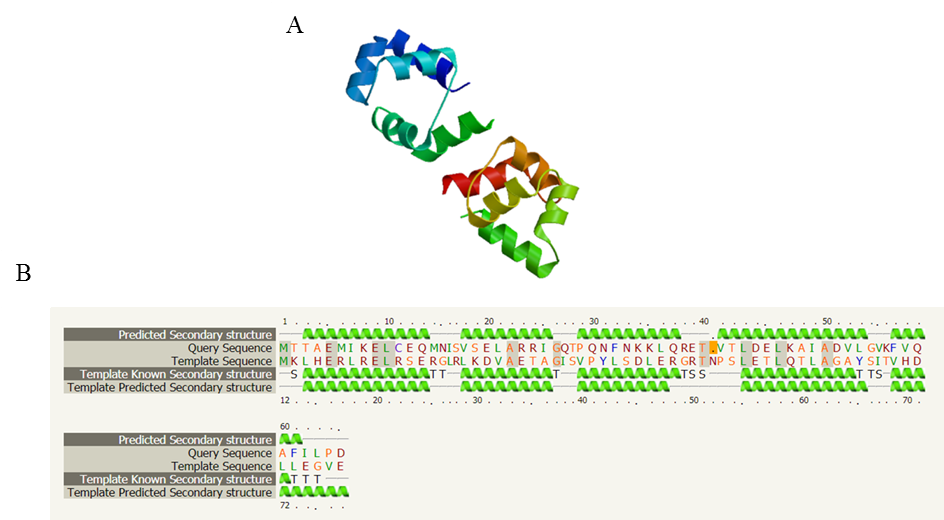


**Supplementary Figure S2. Antitoxin belongs to the Xre family.** (A)SWISS-MODEL-generated 3D structure of antitoxin Xress. (B) PHYRE2 server was used to predict the secondary structure of antitoxin Xress [1]. Protein contains helix-turn-helix (HTH) domain, and its secondary structure is similar to the known ddro secondary structure of *deinococcus geothermalis*.


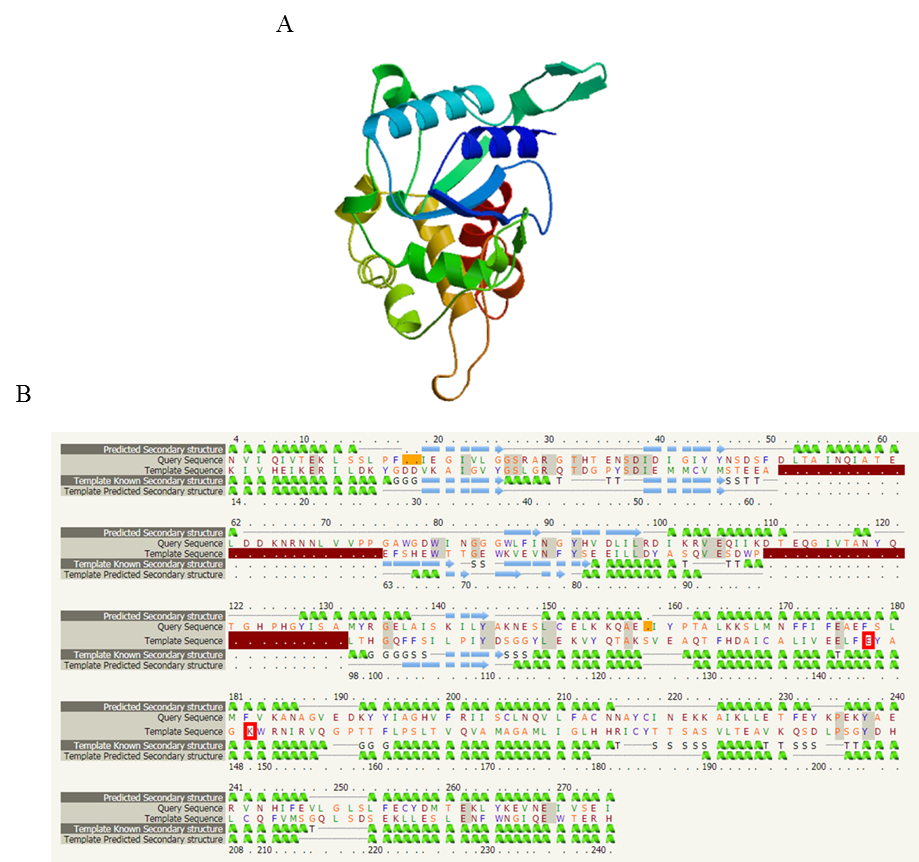


**Supplementary Figure S3. Toxin belongs to the MNT family.** (A) SWISS-MODEL-generated 3D structure of toxin MNTss. (B)The secondary structure of toxin MNTss was predicted using the online PHYRE2 server [1], and the secondary structure of toxin MNTss is similar to the 1kny_A secondary structure from *Staphylococcus aureus*.


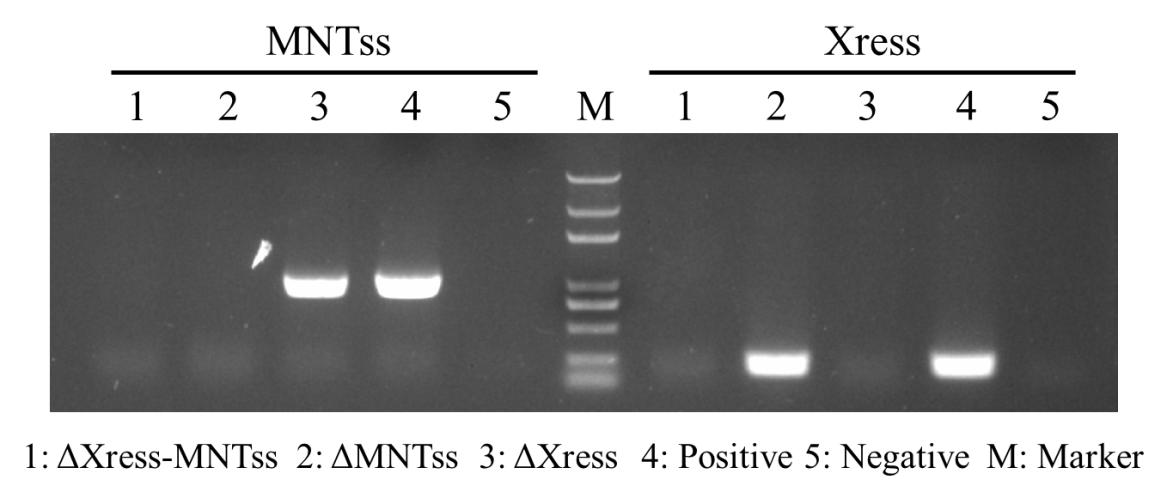


**Supplementary Figure S4. Identification of the deletion strains.** The left panel shows the identification of MNTss in the deletion strains. The right panel shows the identification of Xress in the deletion strains. 1 represents Δ*Xress-MNTss*; 2 represents Δ*MNTss*; 3 represents Δ*Xress*; 4 represents ZY05719 (positive); 5 represents negative; M represents marker.


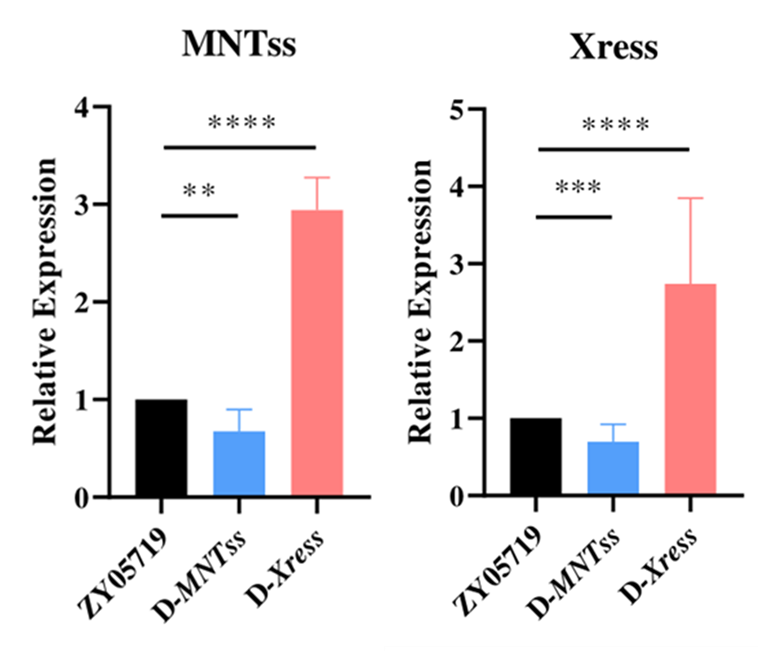


**Supplementary Figure S5. Relative expression level of toxin and antitoxin in point mutants.** qRT-PCR was used to detect the relative expression levels of toxin and antitoxin in point mutants. Unpaired two-tailed Student's *t* test was used for statistical analysis (**, *P*﹤0.01, ***, P < 0.001, ****, *P* < 0.0001).


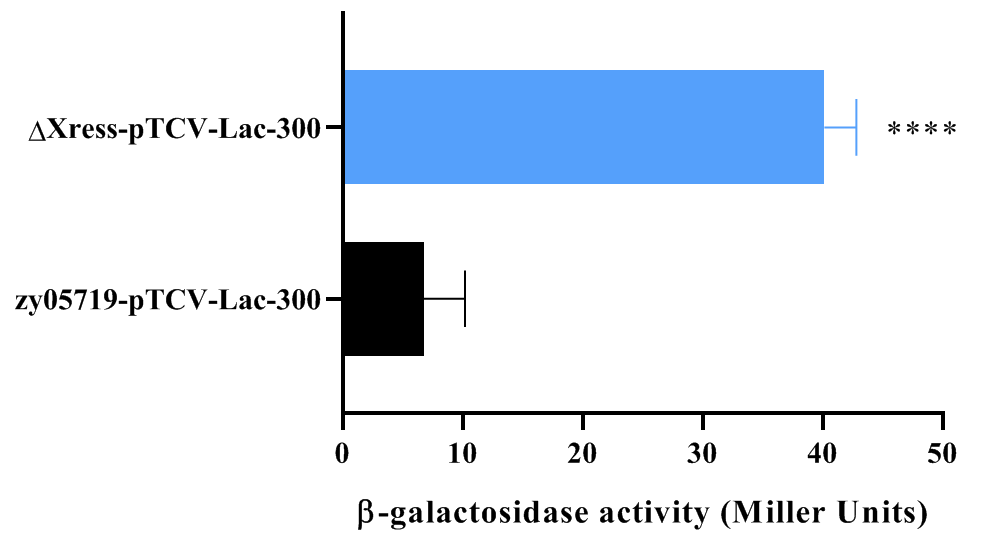


**Supplementary Figure S6. Evaluation of promoter activity in*ΔXress*.** The pTCV-*lac* reporter plasmid (pTCV-*lac*-300), containing the Xress-MNTss promoter sequence, was transferred to Δ*Xress* and ZY05719 to determine β-galactosidase activity. Unpaired two-tailed Student's *t* test was used for statistical analysis (****, *P* < 0.0001).


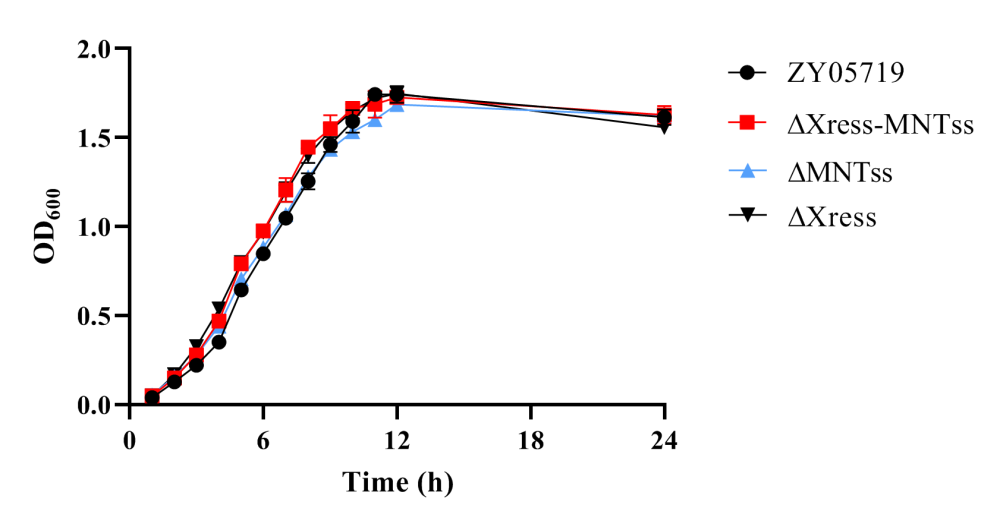


**Supplementary Figure S7.** **Determination of the growth curve of the mutant strains and ZY05719.** Growth curve was constructed based on hourly OD_600_ measurements. The results are indicated as the means±SEM of the results from 3 independent experiments (P﹥0.05).

## Supplementary Tables

**Supplementary Table S1. All primers used in this study.**

| Primer | Sequence^a^ |
| --- | --- |
| Construction of Plasmid |  |
| HisA-F | TGCCGTCACTGCGTCT |
| HisA-R | GCCTGGCAGTTCCCTACTCTC |
| HisA-T2/T3-F | CCGGAATTCTTATATACTTCCATTTCTCAT |
| HisA-T2/T3-R | CCGCTCGAGATGAAGACCTGTTATAAACTC |
| HisA-MNTss-F | CCGCTCGAGATGGTAGGTAATGTTATTCAA |
| HisA-MNTss-R | CGGGGTACCTCATATTTGTTTTCTTTCATC |
| HisA-T6-F | CCGGAATTCATATTATCCTTTCTGGTGCAA |
| HisA-T6-R | CCGCTCGAGGAGATGCAGATTGATCCTATA |
| HisA-pelB-F | TGCCGTCACTGCGTCT |
| HisA-pelB-R | GCCTGGCAGTTCCCTACTCTC |
| HisA-pelB-T2/T3-F | CCGGAATTCTTATATACTTCCATTTCTCAT |
| HisA-pelB-T2/T3-R | CCGCTCGAGATGAAGACCTGTTATAAACTC |
| HisA-pelB-MNTss-F | CCGCTCGAGATGGTAGGTAATGTTATTCAA |
| HisA-pelB-MNTss-R | CGGGGTACCTCATATTTGTTTTCTTTCATC |
| HisA-pelB-T6-F | CCGGAATTCATATTATCCTTTCTGGTGCAA |
| HisA-pelB-T6-R | CCGCTCGAGGAGATGCAGATTGATCCTATA |
| HisA-pelB-Xress-MNTss-F | CCGCTCGAGATGGTAGGTAATGTTATTCAA |
| HisA-pelB-Xress-MNTss-R | CGGGGTACCTTATTCGTTAGATATTTTTAT |
| pTCV-LacZ-F | GTTGAATAACACTTATTCCTATC |
| pTCV-LacZ-R | CTTCCACAGTAGTTCACCACC |
| pTCV-Lac-300-F | ACGAGCGAATCAATGAGCTGA |
| pTCV-Lac-300-R | CAGCACCTCCAAGTTTACGCT |
| pCold^TM^ II-F | ACGCCATATCGCCGAAAGG |
| pCold^TM^ II-R | GGCAGGGATCTTAGATTCTG |
| pCold^TM^ II-Xress-F | CGCGGATCCATGACTACGGCAGAAATGATT |
| pCold^TM^ II-Xress-R | TGCTCTAGATTATTCGTTAGATATTTTTAT |
| pCold^TM^ II-Xress-MNTss-F | CTCGGTACCCTCGAGGGATCCATGACTACGGCAGAAATGATT |
| R | gctacctccgccaccacttccaccgcctccagaacctcctccaccTTCGTTAGATATTTTTATTTC |
| F | ggtggaggaggttctggaggcggtggaagtggtggcggaggtagcATGGTAGGTAATGTTATTCAA |
| pCold^TM^ II-Xress-MNTss-R | AGCAGAGATTACCTATCTAGATCATATTTGTTTTCTTTCATC |
| Construction of deletion strains |  |
| Xress-MNTss-F | ATGACTACGGCAGAAATGATT |
| Xress-MNTss-R | GTTCTACCCGTTTTATATCAC |
| Xress-MNTss-U1-F | AAGAAGAAATTCATCGGAGAA |
| Xress-MNTss-U1-R | TCAGCATTATCCACAGCACCTCCAAGTTTACGC |
| Xress-MNTss-D1-F | GGTAATCAGATTAAATAATCAAAGATACGGAGC |
| Xress-MNTss-D1-R | ATCCTGTTCTGCTAAAGAAAG |
| SacB-SPC-F | GGATAATGCTGAAAACTCCTT |
| SacB-SPC-R | AATCTGATTACCAATTAGAATGAATAT |
| R-Xress-MNTss-1-F | AAGAAGAAATTCATCGGAGAA |
| R-Xress-MNTss-1-R | ATCCTGTTCTGCTAAAGAAAG |
| JC-SacB-Spc-F | ACTATCACGGCTACCACA |
| JC-SacB-Spc-R | TCTTGCCAGTCACGTTACGTT |
| JC-Xress-MNTss-1-F | AAGAACAAATTCCGCACCTTA |
| JC-Xress-MNTss-1-R | AGGTACCATTTGCCGTTCAT |
| Xress-MNTss-U2-F | AAGAAGAAATTCATCGGAGAA |
| Xress-MNTss-U2-R | CTTTGATTATTTACAGCACCTCCAAGTTTACGC |
| Xress-MNTss-D2-F | AAATAATCAAAGATACGGAGC |
| Xress-MNTss-D2-R | ATCCTGTTCTGCTAAAGAAAG |
| R-Xress-MNTss-2-F | AAGAAGAAATTCATCGGAGAA |
| R-Xress-MNTss-2-R | ATCCTGTTCTGCTAAAGAAAG |
| JC-Xress-MNTss-2-F | TGGAGATTGTGGCGATATT |
| JC-Xress-MNTss-2-R | ATAATGAAACGCAAGCGAACT |
| MNTss-F | TGGTAGGTAATGTTATTCAAA |
| MNTss-R | GAGCAGTAGGGTAAATTTCTG |
| MNTss-U1-F | AAGAAGAAATTCATCGGAGAA |
| MNTss-U1-R | TCAGCATTATCCATAAACATCCTCCTTTATTCG |
| MNTss-D1-F | GGTAATCAGATTAAATAATCAAAGATACGGAGC |
| MNTss-D1-R | TGCTACATAATGAAACGCAAG |
| R-MNTss-1-F | AAGAAGAAATTCATCGGAGAA |
| R-MNTss-1-R | TGCTACATAATGAAACGCAAG |
| JC-MNTss-1-F | CTAATCAAGCTGGCGAACAAG |
| JC-MNTss-1-R | CAGGTACCATTTGCCGTTCA |
| MNTss-U2-F | AAGAAGAAATTCATCGGAGAA |
| MNTss-U2-R | CTTTGATTATTTATAAACATCCTCCTTTATTCG |
| MNTss-D2-F | AAATAATCAAAGATACGGAGC |
| MNTss-D2-R | TGCTACATAATGAAACGCAAG |
| R-MNTss-2-F | AAGAAGAAATTCATCGGAGAA |
| R-MNTss-2-R | TGCTACATAATGAAACGCAAG |
| JC-MNTss-2-F | TCTGTGGAGATTGTGGCGATA |
| JC-MNTss-2-R | CCATAGCCGCATCCTAA |
| Xress-F | ATGATTAAAGAACTGTGTGAG |
| Xress-R | TTATTCGTTAGATATTTTTAT |
| Xress-U1-F | GGAGAAGCAATATCTACACAG |
| Xress-U1-R | TCAGCATTATCCTGCCGTAGTCATACAGCACCT |
| Xress-D1-F | GGTAATCAGATTCTAGGTGTCAAGTTTGTGCAG |
| Xress-D1-R | TTTCGGCATATTTTTCAGGTT |
| R-Xress-1-F | GGAGAAGCAATATCTACACAG |
| R-Xress-1-R | TTTCGGCATATTTTTCAGGTT |
| JC-Xress-1-F | TGATTGAATTTATCCGCAAGA |
| JC-Xress-1-R | AGGTACCATTTGCCGTTCAT |
| Xress-U2-F | GGAGAAGCAATATCTACACAG |
| Xress-U2-R | CTTGACACCTAGTGCCGTAGTCATACAGCACCT |
| Xress-D2-F | CTAGGTGTCAAGTTTGTGCAG |
| Xress-D2-R | TTTCGGCATATTTTTCAGGTT |
| R-Xress-2-F | GGAGAAGCAATATCTACACAG |
| R-Xress-2-R | TTTCGGCATATTTTTCAGGTT |
| JC-Xress-2-F | GGCGAGAACGAGCGAATCAAT |
| JC-Xress-2-R | CGCCATTAATCCAATCACC |
| D-MNTss-U1-F | GGAGAAGCAATATCTACACAG |
| D-MNTss-U1-R | TCAGCATTATCCTGCCGTAGTCATACAGCACCT |
| D-MNTss-D1-F | GGTAATCAGATTCTAGGTGTCAAGTTTGTGCAG |
| D-MNTss-D1-R | TTTCGGCATATTTTTCAGGTT |
| R-D-MNTss-1-F | GGAGAAGCAATATCTACACAG |
| R-D-MNTss-1-R | TTTCGGCATATTTTTCAGGTT |
| JC-D-MNTss-1-F | TGATTGAATTTATCCGCAAGA |
| JC-D-MNTss-1-R | AGGTACCATTTGCCGTTCAT |
| D-MNTss-U2-F | AAGAAGAAATTCATCGGAGAA |
| D-MNTss-U2-R | ATTACCTACCAGATAAACATCCTCCTTTATTCG |
| D-MNTss-D2-F | AGGATGTTTATCTGGTAGGTAATGTTATTCAAA |
| D-MNTss-D2-R | TTATAAAGTTTCTCGGTCATA |
| R-D-MNTss-2-F | CTGTGGAGATTGTGGCGATA |
| R-D-MNTss-2-R | ATGATTAACTCTTTCGGCATA |
| JC-D-MNTss-2-F | CAAATCTGGCATCGAAAT |
| JC-D-MNTss-2-R | GTCTGATAATTGGCAGTAACA |
| D-Xress-U1-F | GGAGAAGCAATATCTACACAG |
| D-Xress-U1-R | TCAGCATTATCCTGCCGTAGTCATACAGCACCT |
| D-Xress-D1-F | GGTAATCAGATTCTAGGTGTCAAGTTTGTGCAG |
| D-Xress-D1-R | TTTCGGCATATTTTTCAGGTT |
| R-D-Xress-1-F | GGAGAAGCAATATCTACACAG |
| R-D-Xress-1-R | TTTCGGCATATTTTTCAGGTT |
| JC-D-Xress-1-F | TGATTGAATTTATCCGCAAGA |
| JC-D-Xress-1-R | AGGTACCATTTGCCGTTCAT |
| D-Xress-U2-F | AAGAAGAAATTCATCGGAGAA |
| D-Xress-U2-R | TGCCGTAGTCAGACAGCACCTCCAAGTTTACGC |
| D-Xress-D2-F | GGAGGTGCTGTCTGACTACGGCAGAAATGATTA |
| D-Xress-D2-R | GATTAACTCTTTCGGCATATT |
| R-D-Xress-2-F | CTGTGGAGATTGTGGCGATA |
| R-D-Xress-2-R | ATGATTAACTCTTTCGGCATA |
| JC-D-Xress-2-F | AAGCTGGCGAACAAGGGTCAA |
| JC-D-Xress-2-R | GCCGCCATTAATCCAATCAC |
| qRT-PCR |  |
| Q-Xress-F | CCAGACTCCACAGAATTTCAATAAA |
| Q-Xress-R | AATGCCTGCACAAACTTGAC |
| Q-MNTss-F | GGCGGCGGATGGTTATTTA |
| Q-MNTss-R | AACAATTCCTTGCTCCGTATCT |
| Q-RS04610-F | TTGTGCCGCAAAGAGATACT |
| Q-RS04610-R | CCTTCCACGACATCATCCTAAG |
| parC-F | TGGAGATGCACGGAAACAA |
| parC-R | CTCGATGTCAGCCAAGAGATAG |
| Co-transcription test |  |
| 04620/04625-F | CCGAACTTGCTAGACGTAT |
| 04620/04625-R | GTCTGATAATTGGCAGTAACA |
| 04625/04630-F | AACGAAAGCTTATGCGAATTA |
| 04625/04630-R | ATACAGTGCCATCCATAGCC |
| 04630/04635-F | TATGAGGGCAAACGGACA |
| 04630/04635-R | AAGGGCAATAAGGTAAGATCA |

**References**

1. Kelley LA, Sternberg MJ. Protein structure prediction on the Web: a case study using the Phyre server. Nat Protoc. 2009;4(3):363-71. doi: 10.1038/nprot.2009.2.

**
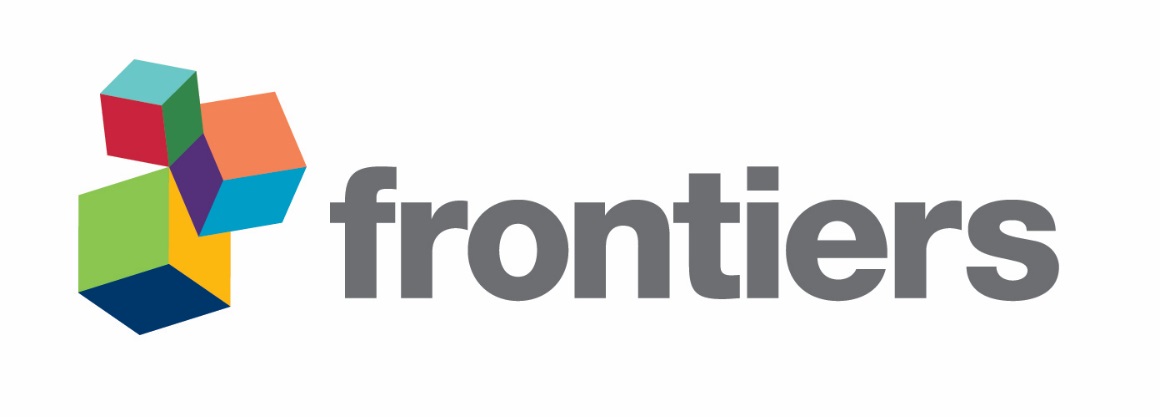
**
